# Evaluation of contrast-induced acute kidney injury using IVIM and DKI MRI in a rat model of diabetic nephropathy

**DOI:** 10.1186/s13244-022-01249-w

**Published:** 2022-06-29

**Authors:** Hongyan Dai, Chun Zhao, Yuxin Xiong, Qian He, Wei Su, Jianbo Li, Ying Yang, Ruyun Lin, Shutian Xiang, Juwei Shao

**Affiliations:** 1 Department of Radiology, The Affiliated Hospital of Yunnan University, NO.176 Qingnian Road, Kunming, 650021 Yunnan China; 2 Department of Endocrinology and Metabolism, The Affiliated Hospital of Yunnan University, Kunming, Yunnan China; 3 Department of Hospital Quality Control, The Affiliated Hospital of Yunnan University, Kunming, Yunnan China

**Keywords:** Contrast-induced acute kidney injury, Diabetic nephropathy, Iodinated contrast media, Intravoxel incoherent motion, Diffusion kurtosis imaging

## Abstract

**Objective:**

To assess the potential of intravoxel incoherent motion (IVIM) and diffusion kurtosis imaging (DKI) in monitoring renal changes in a diabetic nephropathy (DN) rat model with acute kidney injury (CI-AKI) induced by iso-osmotic contrast media (IOCM) and low-osmotic contrast media (LOCM).

**Methods:**

A diabetic nephropathy rat model was established, and the animals were randomly split into the LOCM group and IOCM group (*n* = 13 per group), with iopamidol and iodixanol injection, respectively (4 g iodine/kg). MRI including IVIM and DKI was performed 24 h before contrast medium injections (baseline) and 1, 24, 48, and 72 h after injections. Changes in pure molecular diffusion (*D*), pseudo-diffusion coefficient (D*), perfusion fraction (*f*), mean diffusion (MD), mean kurtosis (MK), serum creatinine (SCr) and urea nitrogen (BUN), histopathology alterations, and α-smooth muscle actin (α-SMA) expression were assessed. Inter-observer agreement was evaluated using the intraclass correlation coefficient (ICC).

**Results:**

Compared against baseline levels, significant decreases in *D*, D*, and *f* were observed in all anatomical kidney compartments after contrast injection (*p* < 0.05). MD in the cortex (CO) and outer medullary (OM) gradually decreased, and MK in OM gradually increased 24–72 h after injection. *D*, D*, *f*, and MD were negatively correlated with the histopathologic findings and α-smooth muscle actin (α-SMA) expression in all anatomical kidney compartments. Inter-observer reproducibility was generally good (ICCs ranging from 0.776 to 0.979).

**Conclusions:**

IVIM and DKI provided noninvasive imaging parameters, which might offer effective detection of CI-AKI in DN.

## Key points

- Nephrotoxicity of IOCM was lower than that of LOCM for diabetic nephropathy rats.
- IVIM and DKI noninvasively detected kidney damage after CI-AKI.
- Functional MRI manifestations were correlated with histopathology findings and α-SMA expression.

## Background

Contrast-induced acute kidney injury (CI-AKI) is a frequently encountered global health problem [1, 2]. Contrast agent-associated acute renal failure is considered one of the most severe complications of CI-AKI [2], and its treatment is very costly. In addition, diabetic nephropathy (DN) as an irreversible disorder is a high-risk factor for CI-AKI [3–5]. Therefore, for DN patients, how to choose the type of contrast agent is particularly important.

At present, the pathogenesis of CI-AKI has not been fully revealed. Serum creatinine (SCr) is the primary parameter used for clinical evaluation of renal function, but it is not sufficiently sensitive to accurately assess the severity and progression of kidney disease [6].

A growing number of noninvasive and non-radiative magnetic resonance imaging (MRI) techniques without utilizing gadolinium contrast media have been applied in diagnosing renal diseases, including intravoxel incoherent motion (IVIM) and diffusion kurtosis imaging (DKI). IVIM estimates tissue microcapillary perfusion by bi-exponential fitting of multi-b-value DWI. As a result, it provides both pure diffusion and pseudo-diffusion (tissue perfusion) information. IVIM assesses renal pathological changes, microcirculatory perfusion, and humoral changes. Liang et al. adopted IVIM to longitudinally observe SD rats with CI-AKI [7]. The changes in *D*, D*, and *f* occurred much earlier than SCr change, which was inconsistent with blood biochemistry results. This indicated that IVIM parameters were sensitive indicators of renal microstructure changes. Wang et al. longitudinally observed a rabbit CI-AKI model using IVIM [8]. It was found that there was a good correlation of histological score with *D* and *f* values. As a new technology, DKI based on non-Gaussian model yields an ultra-high *b* value (> 1000 s/mm^2^). It quantifies the size and direction of water molecule diffusion in tissues, reflects the microstructure of living body [9], and predicts renal damages [10, 11]. Previous studies have shown that DKI can be used to detect the degree of renal fibrosis in patients with IgA nephropathy and diabetic nephropathy [10, 11]. These findings indicated that the renal mean kurtosis (MK) increased with the deterioration of renal function and the progression of renal fibrosis. At present, there is few research on the application of DKI to detect CI-AKI.

This study intended to compare the effects of low- and iso-osmolar contrast media (LOCM and IOCM) on water diffusion, perfusion, and microstructure within renal tissue of DN rats following CI-AKI. Furthermore, we aimed to examine whether IVIM and DKI imaging could noninvasively detect these changes, which were verified by renal pathology and histological α-smooth muscle actin (α-SMA) expression.

## Materials and methods

### Animal model

Ethics approval was obtained from the local hospital ethics committee ((Ethics Committee of Affiliated Hospital of Yunnan University, China).

Twenty-six male Sprague–Dawley (SD) rats (certificate o. SCXK 2015-0002; Kunming, China) weighing 200–250 g were fed a high-fat diet (HFD). Citrate buffer with pH 4.5 was prepared by mixing citric acid and trisodium citrate (0.1 mol/l). Streptozocin (STZ, Sigma-Aldrich, St Louis, MO, USA) solution (3%) was prepared by dissolving 3 mg of STZ in 1 mL of citrate buffer (pH 4.5). After animals were fed with HFD for 21 days, STZ was injected intraperitoneally at doses of 20, 10, and 5 mg/kg/d on the 1st, 3rd, and 5th day, respectively. In addition, 20, 10, 5 mg/kg/d STZ was injected again on the 21st, 23rd, and 25th day, respectively, to induce diabetes in rats [12]. Blood was collected from the rat caudal vein and glucose level was measured. Diabetes in rats was confirmed with a fasting blood glucose (FBG) level of ≥ 16.7 mmol/L. After 6–8 weeks, 24-h urine was collected, and urinary albumin level was determined by the Albumin rat ELISA kit (Sigma), and urinary creatinine level was measured by the picrate method. Combined level of urinary albumin and creatinine was measured to determine whether the DN model was successfully established.

### Experimental groups and procedures

Rats were randomly divided into two groups: LOCM (*n* = 13) and IOCM (*n* = 13). Contrast media were preheated to 37–38 °C, and the two groups of rat were injected with iopamidol-370 and iodixanol-320 (4 g iodine/kg) via the tail vein, respectively. For the control group, rats received the same volume of normal saline injection (i.e., baseline, total 26 rats).

### Biochemistry assessment and histological analysis

Intracardiac blood was withdrawn from rats immediately after MRI scan to examine the renal function indexes, including concentrations of serum creatinine (SCr) and blood urea nitrogen (BUN). Three rats, selected randomly from the two groups, were killed at specific time points (baseline, 1, 24, 48, and 72 h after the injection). Hematoxylin and eosin (H&E) staining and immunohistochemical staining for α-SMA were performed, and the data were assessed by two senior pathologists (with more than 10 years of pathological diagnostic experience). Renal tissue damage was determined using the following scale [13]: injury limited to the glomerular structure (0–1.0), injury extending to the tubule cells (1.1–2.0), and injury including expansion and congestion of the capillaries (2.1–3.0). Immunohistochemical staining for α-SMA was quantified using the Image J software (NIH, Bethesda, MD, USA).

### MRI protocol

**Fig. 1:**
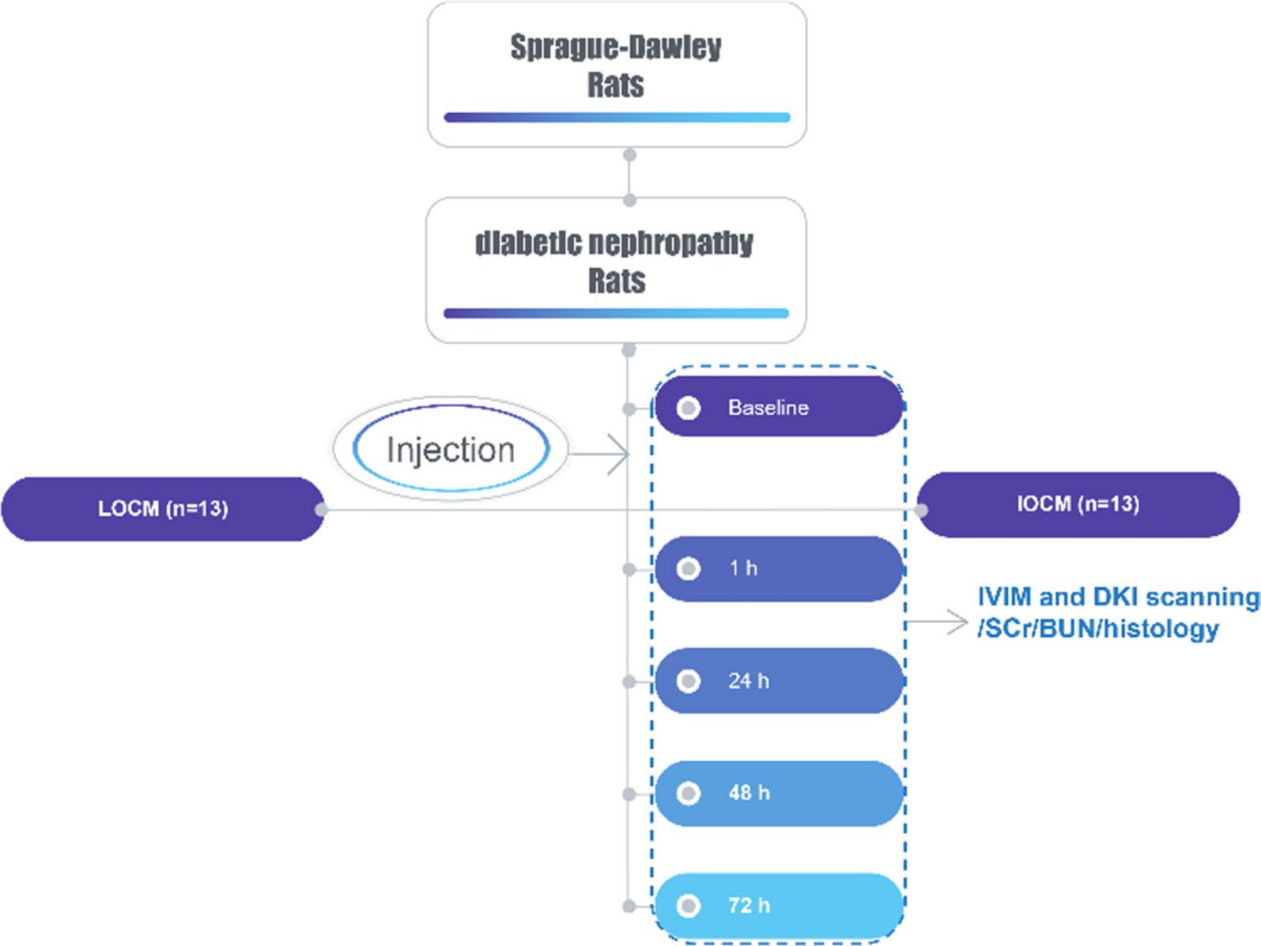
Flowchart

**Fig. 2:**
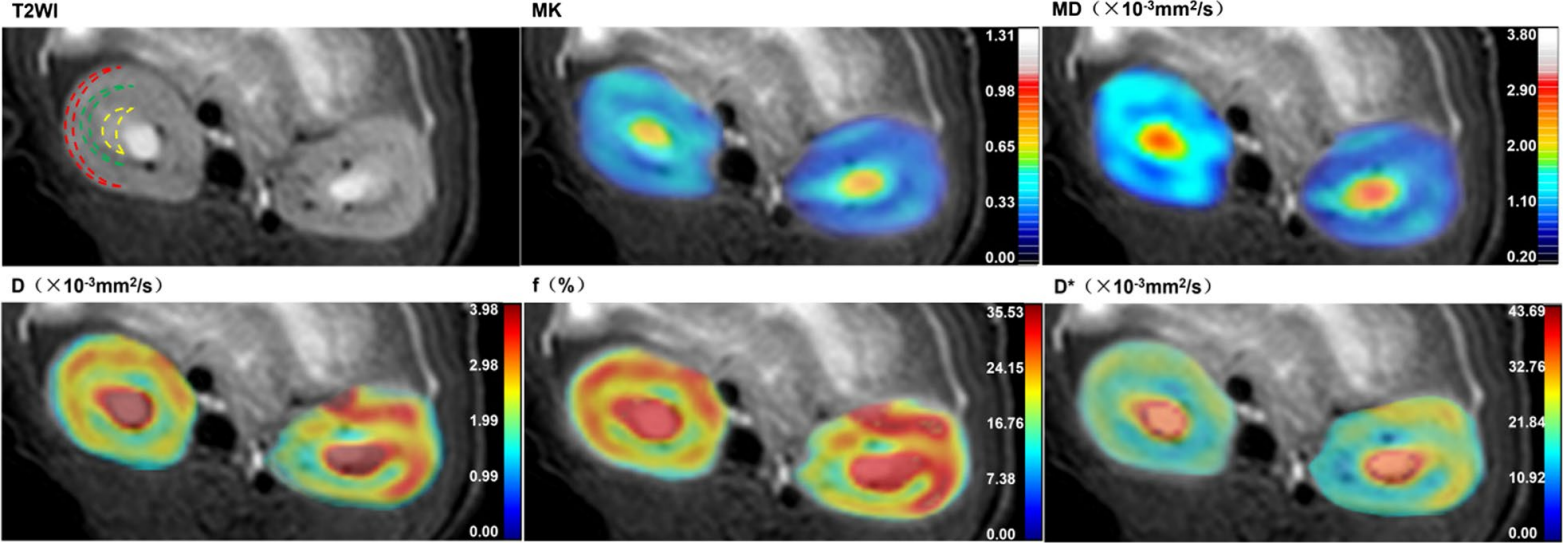
Representative T2-weighted image, MK, MD, *D*, *f*, and D* maps of a rat kidney. T2WI of the kidney showing the location of three ROIs: the CO (the red areas), OM (the green areas), and IM (the yellow areas)

MRI was performed with a 3.0 Tesla scanner (Philips Ingenia, Best, The Netherlands) equipped with a 16-channel (CG-MUC49-H3000-AP) abdominal phased-array surface coil for signal reception (Chenguang Medical Technology Co., Ltd, Shanghai, China). After 8-h fasting, rats were anesthetized with intraperitoneal injection of 10% chloral hydrate (3.0 mL/kg) and immediately scanned in a head‐first prone position. Abdomen of rat was bandaged to reduce the respiratory artifacts during MRI scan. MRI scanning was performed before injection and 1, 24, 48, and 72 h after administration of contrast agents, and pre-injection data were used as baseline values (Fig. 1). MRI sequences applied included axial T2-weighted imaging, axial IVIM, and axial DKI (Fig. 2).

IVIM adopted the following parameters: repetition time (TR)/echo time (TE), 1262/94 ms; slice thickness/gap, 2.0/0.5 mm; matrix, 110 × 110; field of view (FOV), 24 × 24 cm^2^; and nine *b* values, 0, 20, 50, 80, 100, 200, 400, 600, and 800 s/mm^2^.

DKI was acquired using the following parameters: TR/TE, 1555/92 ms; slice thickness/gap, 2.0/0.5 mm; matrix, 110 × 110; FOV, 24 × 24 cm^2^; and four *b* values, 0, 500, 1000, and 1500 s/mm^2^.

### IVIM and DKI image analyses

Quantitative parameters including pure molecular diffusion (*D*), pseudo-diffusion coefficient (D*), perfusion fraction (*f*), mean diffusion (MD), and mean kurtosis (MK) were obtained by processing IVIM and DKI images using MITK software (www.mitk.org). All IVIM and DKI images were independently analyzed by two physicians with more than 5 years of diagnostic experience in abdominal MRI. Final score was the average of the scores obtained by the two observers. Freehand regions of interest (ROIs) were placed in the cortex (CO), the outer medulla (OM), and the inner medulla (IM) using MITK software (Fig. 2).

### Statistical analysis

The data were expressed as mean ± standard deviation (SD). One-way analysis of variance (ANOVA) and Tukey’s post hoc test were used for comparisons across multiple groups. Repeated-measure ANOVA and Bonferroni post hoc test for multiple comparisons were used to analyze within-group changes of MRI parameters. Correlation analysis between different groups was performed using Spearman’s correlation test. Intraclass correlation coefficient (ICC) was used in the analysis of inter-observer agreement between the two radiologists. As given by Bobos et al. [14], an ICC value < 0.40 indicated poor inter-rater agreement; 0.40–0.59 indicated fair agreement; 0.60–0.74 indicated good agreement; and 0.75–1.00 indicated excellent agreement. All statistical analyses were conducted using SPSS version 26.0 (SPSS, Chicago, IL, USA). *p* < 0.05 (two-tailed) indicated a statistical significance.

## Results

### Progressive changes in IVIM parameters for CI-AKI

**Fig. 3:**
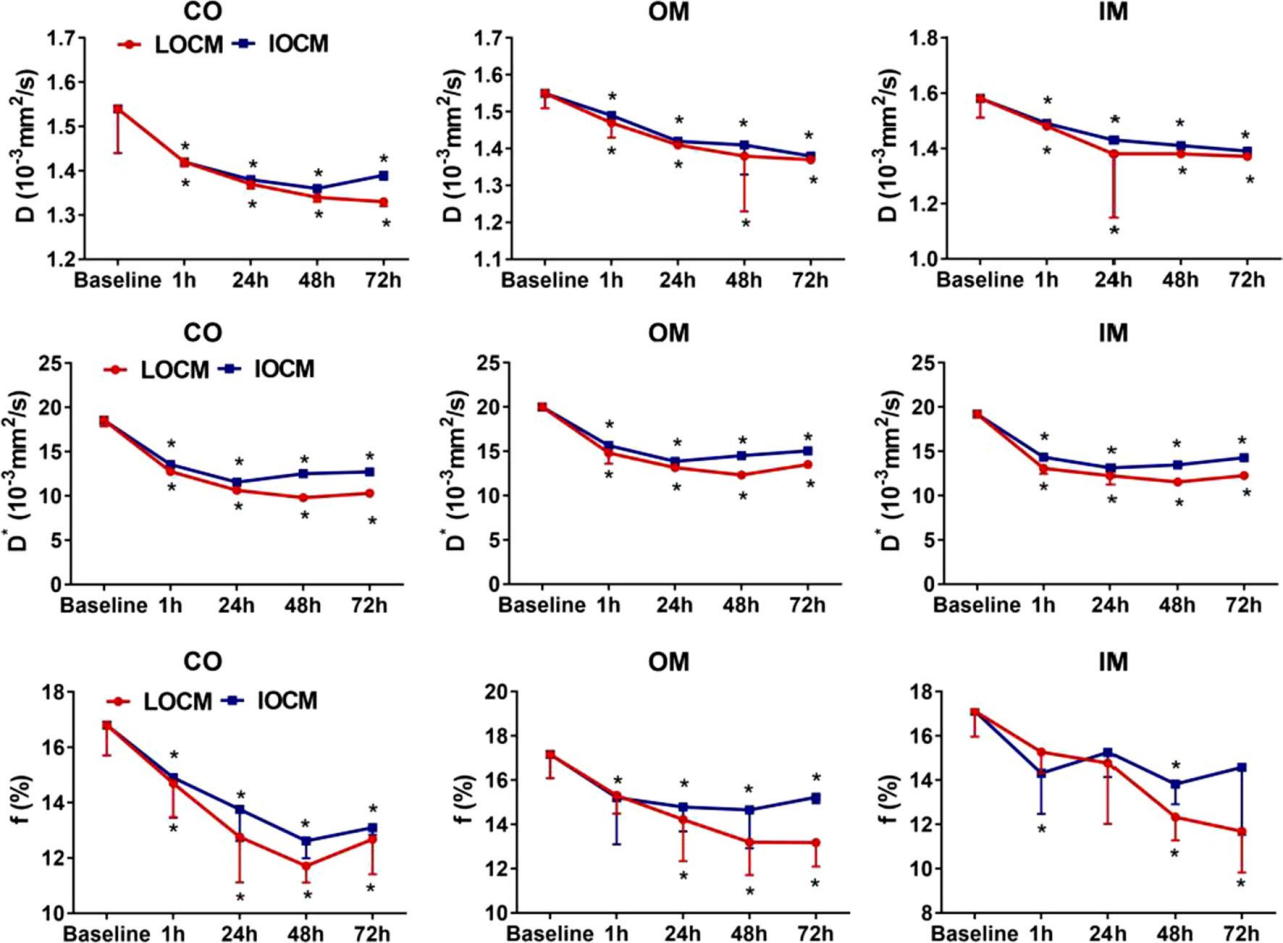
The temporal changes of *D*, D*, and *f* in all the anatomical layers. *Represents *p* < 0.05 versus baseline

Compared against baseline values, *D* in all renal anatomical compartments decreased in both groups after contrast injection (Table 1 and Fig. 3). *D* was significantly decreased in the CO and OM of LOCM group (*p* = 0.003 and *p* < 0.001, respectively), and CO and OM of IOCM group (*p* = 0.006 and *p* < 0.001, respectively) 1 h after injection. *D* was significantly decreased at 48-h in the IM of LOCM and IOCM groups (both *p* < 0.001) compared against the baseline values. Cortical *D* in the CO of IOCM group reached the lowest level at 48-h (*p* < 0.001) and recovered at 72-h (*p* < 0.001), while the value of the other groups showed a downward trend at 72-h.

**Table 1:** Mean IVIM parameters at different time points during the course of CI-AKI

	Group	Baseline	1 h	24 h	48 h	72 h
*D* (× 10^–3^ mm^2^/s)						
CO	LOCM	1.54 ± 0.10	1.42 ± 0.01*	1.37 ± 0.01*	1.34 ± 0.01*	1.33 ± 0.01*
	IOCM		1.42 ± 0.01*	1.38 ± 0.01*	1.36 ± 0.02*	1.39 ± 0.01*
OM	LOCM	1.55 ± 0.04	1.47 ± 0.04*	1.41 ± 0.01*	1.38 ± 0.15*	1.37 ± 0.01*
	IOCM		1.49 ± 0.01*	1.42 ± 0.01*	1.41 ± 0.08*	1.38 ± 0.01*
IM	LOCM	1.58 ± 0.07	1.48 ± 0.01*	1.38 ± 0.23	1.38 ± 0.01*	1.37 ± 0.01*
	IOCM		1.49 ± 0.01*	1.43 ± 0.01	1.41 ± 0.01*	1.39 ± 0.02*
D* (× 10^–3^ mm^2^/s)						
CO	LOCM	18.55 ± 0.68	12.77 ± 0.16*	10.64 ± 0.02*	9.80 ± 0.05*	10.31 ± 0.26*
	IOCM		13.55 ± 0.29*	11.56 ± 0.28*	12.50 ± 0.25*	12.71 ± 0.20*
OM	LOCM	20.02 ± 0.26	14.83 ± 1.21*	13.15 ± 0.02*	12.33 ± 0.21*	13.50 ± 0.29*
	IOCM		15.65 ± 0.12*	13.87 ± 0.04*	14.50 ± 0.24*	15.05 ± 0.46*
IM	LOCM	19.19 ± 0.31	13.08 ± 0.62*	12.23 ± 1.00*	11.53 ± 0.24*	12.26 ± 0.14*
	IOCM		14.34 ± 0.04*	13.13 ± 0.18*	13.46 ± 0.18*	14.28 ± 0.28*
*f* (%)						
CO	LOCM	16.80 ± 1.10	14.70 ± 1.22*	12.76 ± 1.64*	11.71 ± 0.60*	12.68 ± 1.27*
	IOCM		14.90 ± 1.46	13.76 ± 1.15*	12.62 ± 0.63*	13.10 ± 0.26*
OM	LOCM	17.16 ± 1.08	15.31 ± 0.83	14.22 ± 1.88*	13.20 ± 1.49*	13.18 ± 1.08*
	IOCM		15.21 ± 2.12*	14.79 ± 1.10*	14.65 ± 1.74*	15.23 ± 0.27*
IM	LOCM	17.12 ± 1.16	15.27 ± 0.95	14.77 ± 2.75	12.33 ± 1.05*	11.69 ± 1.86*
	IOCM		14.31 ± 1.84*	15.25 ± 1.12	13.82 ± 0.91*	14.58 ± 3.05
MD (× 10^–3^ mm^2^/s)						
CO	LOCM	3.20 ± 0.16	3.18 ± 0.03	3.10 ± 0.03	2.86 ± 0.13*	2.76 ± 0.20*
	IOCM		3.19 ± 0.06	2.91 ± 0.006	2.80 ± 0.03*	2.77 ± 0.09*
OM	LOCM	2.29 ± 0.24	2.37 ± 0.02	2.25 ± 0.03	2.06 ± 0.05	1.77 ± 0.10*
	IOCM		2.36 ± 0.17	2.33 ± 0.17	2.26 ± 0.15	1.88 ± 0.07*
IM	LOCM	2.36 ± 0.26	2.39 ± 0.22	2.33 ± 0.24	2.24 ± 0.37	2.24 ± 0.29
	IOCM		2.31 ± 0.24	2.31 ± 0.14	2.11 ± 0.19	2.08 ± 0.24
MK						
CO	LOCM	0.76 ± 0.03	0.74 ± 0.01	0.76 ± 0.04	0.78 ± 0.05	0.85 ± 0.12
	IOCM		0.75 ± 0.06	0.75 ± 0.03	0.76 ± 0.10	0.77 ± 0.09
OM	LOCM	0.81 ± 0.01	0.77 ± 0.02*	0.84 ± 0.04	0.86 ± 0.02	0.90 ± 0.04*
	IOCM		0.78 ± 0.03*	0.82 ± 0.06	0.85 ± 0.08	0.86 ± 0.04
IM	LOCM	0.80 ± 0.14	0.71 ± 0.02	0.72 ± 0.03	0.76 ± 0.10	0.82 ± 0.08
	IOCM		0.71 ± 0.02	0.71 ± 0.04	0.74 ± 0.07	0.78 ± 0.10

*Represents *p* < 0.05 versus baseline

Compared against the baseline data, D* in each anatomical compartment of the kidney was significantly decreased 1 h after injecting contrast agents in both groups (Table 1 and Fig. 3). D* in each anatomical compartment of LOCM group reached the lowest value at 48-h (*p* < 0.001) and showed an upward trend at 72-h (*p* < 0.001). D* in each anatomical compartment of IOCM group reached the lowest value at 24-h (*p* < 0.001) and showed an upward trend at 48-h (*p* < 0.001).

Compared against the baseline levels, *f* in each anatomical compartment of the kidney was significantly reduced 1 h after contrast injection in both groups (Table 1 and Fig. 3). Cortical f in the CO of LOCM group reached the lowest value at 48-h (*p* < 0.001) and showed a trend of recovery at 72-h (*p* < 0.001). Medullary f in the OM and IM of LOCM group also showed a decreasing trend after 72 h (*p* < 0.001). Additionally, *f* in each anatomical compartment of IOCM group reached the lowest value at 48-h (*p* < 0.001, *p* = 0.039, and *p* = 0.001, respectively) and showed an increasing trend at 72-h (*p* < 0.001, *p* = 0.010, and *p* = 0.113, respectively).

### Progressive changes of DKI parameters for CI-AKI

**Fig. 4:**
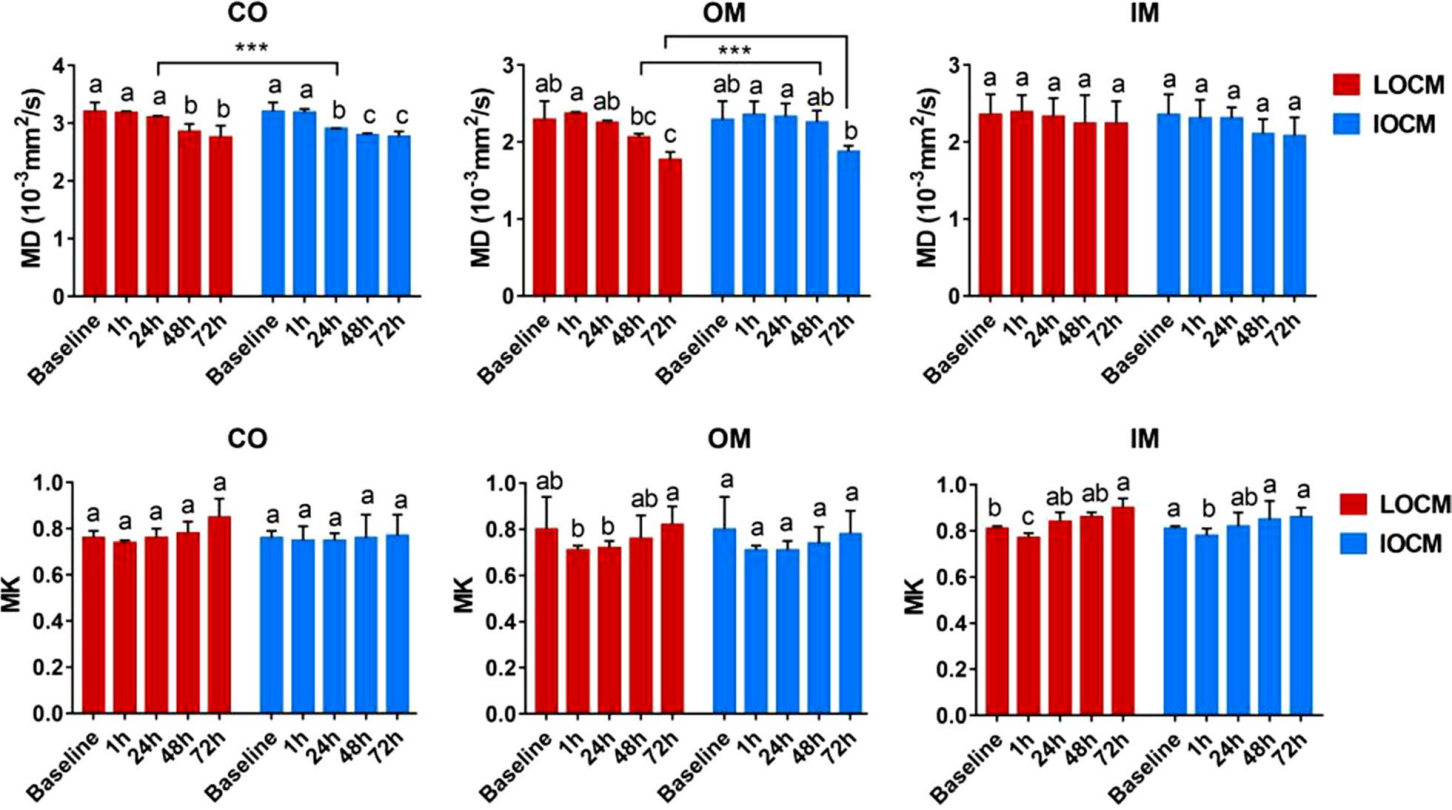
The comparison of MD and MK at all anatomical layers at each time point was compared between the LOCM group and the IOCM group. Different lowercase letters on the column represent significant differences between groups at the 0.05 level. ***Represents a significant difference between groups at the level of 0.001

MD was slightly increased in the OM and IM of the LOCM group (*p* = 1.000), and OM of IOCM group (*p* = 1.000) 1 h after contrast injection compared against baseline values (Table 1 and Fig. 4). MD was slightly decreased in the CO of LOCM group (*p* = 1.000) and CO and IM of IOCM group (*p* = 1.000) at 1-h. After injection of contrast media for 24–72 h, MD in each anatomical compartment was continuously decreased in both groups.

At 1-h after injection, MK was slightly decreased in each anatomical compartment of LOCM group (*p* = 0.995, *p* = 0.006, and *p* = 0.570, respectively) and IOCM group (*p* = 1.000, *p* = 0.035, and *p* = 0.617, respectively) and returned to baseline level at 24–72 h (Table 1 and Fig. 4).

### Histological analysis

**Fig. 5:**
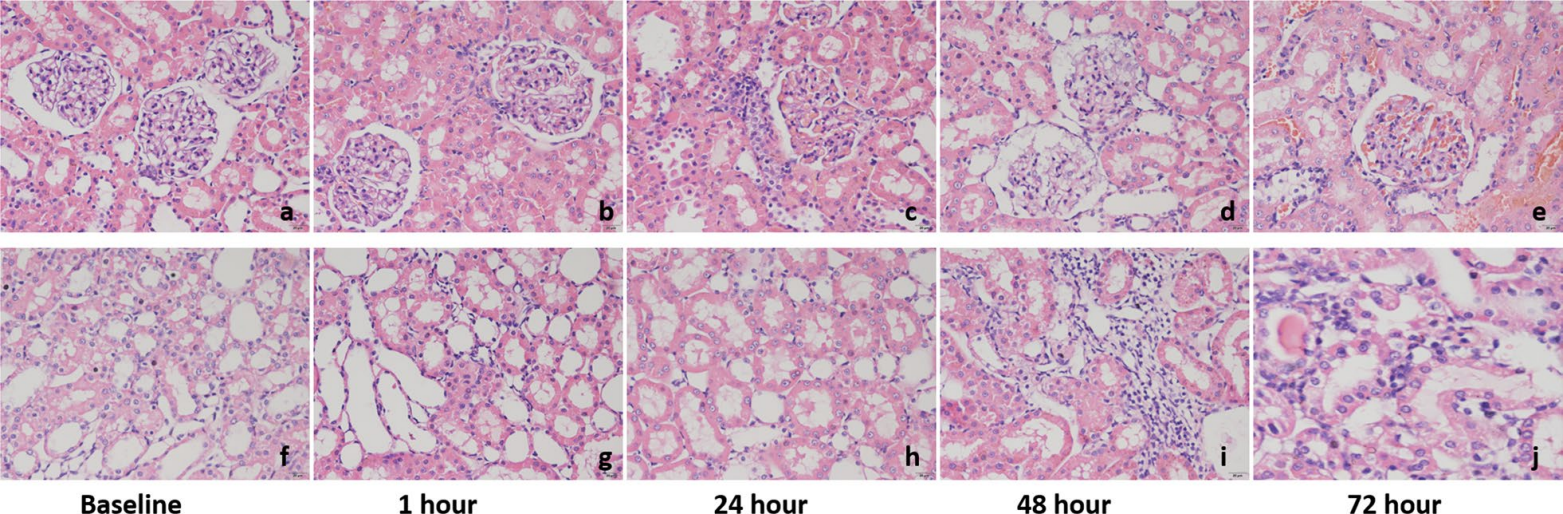
Pathological images of HE staining of rat kidney at different time points. Representative pathologic images: **a**, **f** Diabetic nephropathy rats without contrast injection. **b**–**e**, **g**–**j** Diabetic nephropathy rats injected with contrast agent (**b**, **g**: 1 h; **c**, **h**: 24 h; **d**, **i**: 48 h; **e**, **j**: 72 h)

Pathological sections at each time point showed focal and segmental hyperplasia of glomerular mesangial cells, increased mesangial matrix deposition, disordered arrangement of renal tubular epithelial cells, and cell edema in all DN rats (Fig. 5).

Dilatation of renal tubules, partial swelling of glomerular cells, and hyperemia in the glomerulus were observed 1 h after injection. At 24-h, a large number of erythrocytes were detected in the glomerulus, and abundant lumen proteins were observed in the renal tubule. At 48-h, degeneration of the glomerular basement membrane and infiltration of inflammatory cells around renal tubules were observed. At 72-h, severe glomerular congestion, degeneration, and cell edema were observed, and urinary proteins were detected in renal tubules.

Compared against baselines, LOCM and IOCM groups showed significantly increased injury scores at 1-, 24-, 48-, and 72-h (*p* = 0.012, *p* = 0.010, *p* < 0.001, and *p* < 0.001; *p* = 0.034, *p* = 0.017, and *p* < 0.001, respectively) (Table 2).

**Table 2:** The change of kidney pathological scores and α-SMA expression scores of DN rat before and after injection of contrast agent

	Group	Baseline	1 h	24 h	48 h	72 h	(F) *P*
Pathological scores	LOCM	1.02 ± 0.15	1.40 ± 0.14*	1.40 ± 0.13*	1.78 ± 0.15*	1.87 ± 0.14*	(16.049) 0.001
	IOCM		1.27 ± 0.15	1.33 ± 0.10*	1.43 ± 0.12*	1.80 ± 0.14*	(11.235) 0.004
	*P*	–	0.145	0.341	0.001	0.426	
α-SMA expression scores	LOCM	3.43 ± 0.20	3.79 ± 0.51	3.73 ± 0.51	4.23 ± 0.17*	4.19 ± 0.38*	(8.663) 0.018
	IOCM		3.44 ± 0.18	3.60 ± 0.25	3.73 ± 0.30	4.02 ± 0.26*	(4.721) 0.060
	*P*	–	0.187	0.642	0.013	0.424	

*Represents *p* < 0.05 versus baseline

### α-SMA immunohistochemistry staining results

**Fig. 6:**
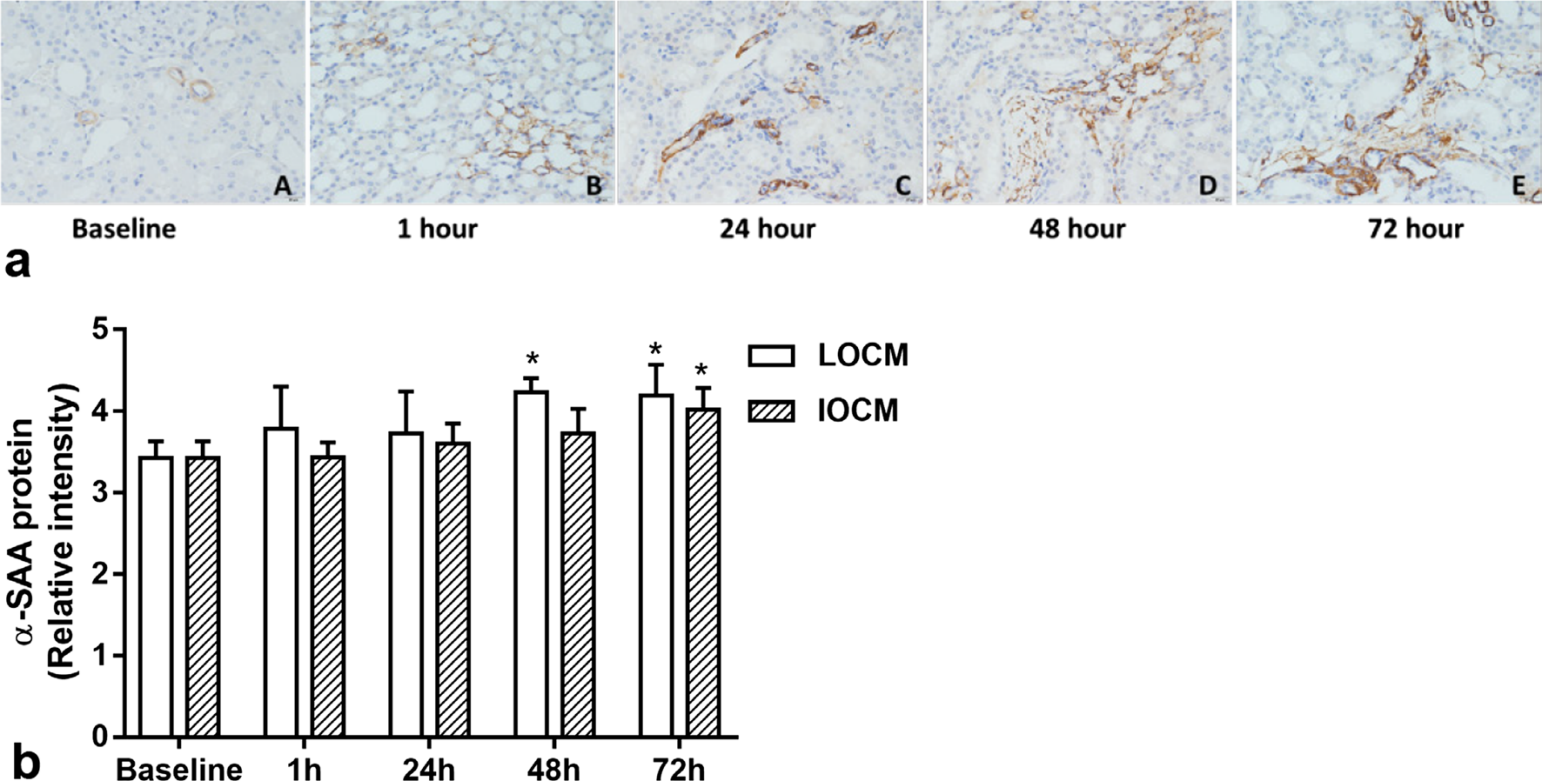
**a** Expression of α-SMA in rat kidney at different time points. **b** The histogram of expression level of α-SMA in kidney of rats before and after injection of contrast agent. *Represents *p* < 0.05 versus baseline

The expression level of α-SMA in the renal interstitium of the two groups of rats gradually increased (Fig. 6). Quantitative analysis showed that the difference in α-SMA of the LOCM group against baseline at 48-h and 72-h was statistically significant (*p* = 0.006 and *p* = 0.003, respectively), and the difference comparing the α-SMA of IOCM group against baseline at 72-h was statistically significant (*p* = 0.014) (Table 2).

### Blood biomarkers

After injecting contrast agents, SCr and BUN in both groups increased compared to baseline values (Table 3).

**Table 3:** Blood biochemical examinations of DN rats in two groups

Group	Time	SCr (μmol/L)	BUN (mmol/L)
	Baseline	26.69 ± 2.12	12.66 ± 2.16
LOCM	1 h	27.33 ± 1.53	12.71 ± 1.47
	24 h	30.00 ± 4.00	13.76 ± 2.65
	48 h	32.67 ± 4.04	14.96 ± 3.76*
	72 h	35.00 ± 3.61*	14.28 ± 2.77*
IOCM	1 h	27.00 ± 2.76	13.51 ± 3.75
	24 h	33.00 ± 9.54	14.35 ± 2.45
	48 h	33.67 ± 2.08	14.57 ± 1.36
	72 h	28.33 ± 2.08	12.81 ± 3.89*

*Represents *p* < 0.05 versus baseline

### Correlation of MRI parameters with pathological scores

As shown in Table 4, a significantly negative correlation was established between kidney injury score and MD in IM. Also, a negative correlation of kidney injury score with *D* in all anatomical compartments, D* in all renal anatomical compartments, *f* in CO and OM, and MD in CO and OM was observed. Kidney injury score showed a low correlation with MK in OM.

**Table 4:** Correlation analyses between the MRI parameters and laboratory test index, pathological score, and immunohistochemical expression

Spearman’s correlation (*r*, *P*)	*D* (× 10^–3^ mm^2^/s)			D* (× 10^–3^ mm^2^/s)			*f* (%)			MD (× 10^–3^ mm^2^/s)			MK		
	CO	OM	IM	CO	OM	IM	CO	OM	IM	CO	OM	IM	CO	OM	IM
SCr (μmol/L)	− 0.624, < 0.001	− 0.448, < 0.001	− 0.543, < 0.001	− 0.581, < 0.001	− 0.515, < 0.001	− 0.537, < 0.001	− 0.462, < 0.001	− 0.363, < 0.001	− 0.315, < 0.001	− 0.491, < 0.001	− 0.367, < 0.001	− 0.169, 0.051	0.118, 0.172	0.276, 0.001	0.137, 0.113
BUN (mmol/L)	− 0.234, 0.007	− 0.14, 0.11	− 0.194, 0.026	− 0.216, 0.013	− 0.196, 0.025	− 0.189, 0.03	− 0.15, 0.094	− 0.107, 0.233	− 0.093, 0.303	− 0.194, 0.024	− 0.094, 0.28	− 0.076, 0.384	0.02, 0.822	0.089, 0.302	0.181, 0.036
Pathology scores	− 0.784, < 0.001	− 0.623, < 0.001	− 0.775, < 0.001	− 0.674, < 0.001	− 0.616, < 0.001	− 0.583, < 0.001	− 0.568, < 0.001	− 0.510, < 0.001	− 0.273, 0.002	− 0.761, < 0.001	− 0.576, < 0.001	− 0.807, < 0.001	0.188, 0.029	0.452, < 0.001	0.268, 0.002
α-SMA	− 0.742, < 0.001	− 0.617, < 0.001	− 0.574, < 0.001	− 0.565, < 0.001	− 0.543, < 0.001	− 0.461, < 0.001	− 0.813, < 0.001	− 0.823, < 0.001	− 0.707, < 0.001	− 0.756, < 0.001	− 0.824, < 0.001	− 0.737, < 0.001	0.153, 0.077	0.399, < 0.001	0.209, 0.015

### Correlation of MRI parameters with α-SMA expression

As shown in Table 4, a significantly negative correlation was established between the α-SMA expression level and *f* in CO, OM, and MD in OM. Also, a good negative correlation of α-SMA expression level with *D* in all anatomical compartments, D* in CO and OM, *f* in IM, and MD in CO and IM was established. The α-SMA expression level showed a low correlation with D* in IM and MK in OM.

### Correlation of MRI parameters with blood biomarkers

As shown in Table 4, a negative correlation of SCr level with *D* in CO and IM and D* in all anatomical compartments was established. Also, a negative correlation of SCr level with *D* in OM, *f* in all anatomical compartments, and MD in CO and OM was detected. On the other hand, no correlation was established between BUN level and MRI parameters.

### Reproducibility

Inter-observer and intra-observer agreements were excellent in all measured quantitative parameters for each anatomical compartment (Table 5).

**Table 5:** Inter-observer and intra-observer reliability estimated by intraclass correlation coefficient (ICC)

	ICC (95% IC)					
	Intra-observer			Inter-observer		
	CO	OM	IM	CO	OM	IM
*D*	0.956 (0.909–0.979)	0.847 (0.66–0.935)	0.828 (0.634–0.925)	0.961 (0.871–0.989)	0.979 (0.916–0.994)	0.975 (0.919–0.993)
D*	0.979 (0.952–0.991)	0.808 (0.602–0.914)	0.797 (0.578–0.91)	0.996 (0.986–0.999)	0.891 (0.727–0.959)	0.723 (0.236–0.918)
*f*	0.776 (0.532–0.902)	0.875 (0.738–0.942)	0.810 (0.633–0.906)	0.825 (0.429–0.954)	0.841 (0.514–0.955)	0.978 (0.916–0.994)
MD	0.927 (0.851–0.965)	0.851 (0.704–0.928)	0.826 (0.643–0.919)	0.976 (0.929–0.992)	0.951 (0.855–0.984)	0.918 (0.718–0.978)
MK	0.812 (0.633–0.908)	0.931 (0.851–0.969)	0.939 (0.863–0.974)	0.937 (0.818–0.979)	0.968 (0.902–0.99)	0.753 (0.333–0.923)

All the ICCs reached significant (*p* < 0.01)

## Discussion

The goal of this study was to compare the adverse effects of LOCM and IOCM on renal water diffusion, perfusion, and tissue microstructure in DN rats diagnosed by IVIM and DKI. MRI findings were substantiated by renal histopathology and α-SMA immunostaining results.

After contrast injection, pure molecular diffusion (*D*) values in each anatomical compartment of the kidney in both groups decreased gradually from 1 h compared against baseline, indicating contrast-induced renal vascular hypoperfusion [15], which was further verified by H&E staining. Moreover, swollen cells appeared in the proximal convolved tubules and parts of the glomerulus at the corresponding time points, resulting in increased resistance to fluid flow in the renal tubules [16]. Another finding was that the lowest values of *D* in the IOCM group were noted at 48-h in CO and 72-h in IM and OM, and the lowest *D* values in the LOCM group were recorded at 48-h in CO and 72-h in IM and OM. This phenomenon suggested that the cortex recovered more rapidly than the medulla after kidney injury and that the medulla was highly sensitive to hypoxia [17, 18]. These manifestations led to severe ischemia and hypoxia injury [13], and the recovery time was prolonged.

From 1 h after the injection of contrast agent, the perfusion-related parameters including pseudo-diffusion coefficient (D*) and perfusion fraction (*f*) declined gradually, suggesting that the volume of cortical and medulla fluid decreased due to renal tubule response and glomerular vasoconstriction [19]. At 1 h, the decrease in D* was more obvious than that in *f*, indicating that vasoconstriction occurred before fluid volume decrease. The recovery time point of D* was earlier than that of *f*. Thus, we speculated that the recovery of D* was related to vascular dilation, and the subsequent increase in *f* indicated an enlargement in vascular fluid volume (i.e., blood volume) [20], which was caused by vascular dilation. The reason for this mechanism may be that D* depends largely on vasoconstriction or vasodilatation, while the vasodilatation may increase the capillary pressure, leading to continuous rise of blood volume. Subsequently, with the increase in effective filtration pressure, the capillary blood volume and blood flow of the kidney are gradually restored to the original levels. Thus, the reversion of blood volume could be attributed to the recovery of vasodilation [21, 22]. These findings suggested that parameter D* was more sensitive than others, which was consistent with the observations by Zhang et al. [23].

MK value in the cortex was lower than that in the ipsilateral medulla. This might be related to the complex structure of the renal medulla, especially renal tubules and collecting tubules, which affected the diffusion limitation of water molecules and made the diffusion distribution deviate from the Gaussian form. This finding was consistent with the previous results by Huang et al. [24]. Cortical MD value was higher than that of ipsilateral medulla, which might be due to fewer diffusion barriers, richer blood perfusion, and more active movement of water molecules in the cortex compared to medulla [10]. When the contrast agent was injected for 1 h, MD in OM in both groups showed an upward trend, and MK in each renal anatomical compartment in both groups showed a downward trend. This phenomenon could be attributed to the direct toxic effect of iodine as a contrast agent, triggering renal tubular necrosis and hindering the diffusion of water molecules, thereby causing tubular structure damage. Simultaneously, the cortical and medullary microstructures were damaged, leading to free diffusion of water molecules, which was deviated from the Gaussian model [25]. At the time point of 24–72 h, MD in each renal anatomical compartment in both groups decreased, and MK increased probably due to inflammatory cell infiltration, leading to fibrous formation [26], limited water molecule diffusion within renal parenchyma, and progressive fibrosis of renal cortex and medulla [27].

The lowest *D* in CO, lowest D* in each anatomical compartment, and lowest f in IM and OM of the IOCM group were detected earlier and greater than those of the LOCM group, suggesting that the renal toxicity in LOCM group was more severe compared with IOCM group, as described previously [28, 29]. In this study, 48 h after injecting contrast media, the pathological score and the expression level of α-SMA in the LOCM group were distinctly higher than those in the IOCM group (both *p* < 0.05). Combined with MRI findings, the pathological and immunohistochemical analyses suggested that the osmotic pressure of the contrast agent might be the main factor contributing to nephrotoxicity. Long-term nephrotoxicity in the LOCM group was more severe compared with the IOCM group, manifesting as severe renal damage and a higher degree of renal fibrosis in DN rats [30, 31]. This effect might be explained by the fact that the osmotic pressure of the contrast agent became the leading cause of nephrotoxicity under the background condition of DN, and the original foundation of kidney injury accelerated the occurrence and development of comparative kidney injury [32, 33]. Therefore, osmotic pressure exerted a critical influence on renal function in DN patients, and the application of an iso-osmolar contrast medium might be beneficial for these patients.

In addition, the changing trends of parameters in the two groups at other time points were similar, indicating that the mechanism underlying kidney injury induced by two different contrast agents was similar. The current study showed that SCR and BUN were not sensitive indicators of kidney injury, and the changes in *D*, D*, *f*, MD, and MK occurred much earlier than the changes in blood SCR and BUN [7]. Herein, we observed that most of the IVIM and DKI parameters were correlated with SCR, pathological score, and α-SMA expression. Therefore, we speculated that IVIM and DKI were effective tools in differentiating the changes in renal microcirculation and diagnosing CI-AKI.

In the present study, no significant correlation was established between certain MRI parameters and the two laboratory indexes. This might be due to slight pathological alterations of the tissue structure in early CI-AKI and strong renal compensatory ability, which were not sufficient to cause significant changes in the corresponding laboratory examination results.

Nevertheless, the current study had some limitations. First, the influence of viscosity on renal structure and function was not excluded. Therefore, in future studies, we shall focus on the effect of contrast media viscosity on renal function. Second, the observation period was relatively short, and long-term effects of the contrasts on kidney of diabetic nephropathy could be different. Third, there was no normal control group of animals without injection included. However, this limitation did not influence our observations.

In conclusion, most parameters of IVIM and DKI were significantly correlated with the pathological score and α-SMA expression level. Thus, IVIM and DKI might reflect the effects of iodinated contrast media with a different osmolarity on renal water diffusion, perfusion, and tissue microstructure.

## Data Availability

All data and materials are available within the article.

## Abbreviations

CI-AKI: Contrast-induced acute kidney injury
DKI: Diffusion kurtosis imaging
DN: Diabetic nephropathy
IOCM: Iso-osmolar contrast medium/media
IVIM: Intravoxel incoherent motion
LOCM: Low-osmolar contrast medium/media
ROI: Region of interest
TE: Echo time
TR: Repetition time
